# Demographics, facilitators, and barriers among predominantly heated yoga users: a survey of the largest U.S. yoga studio brand

**DOI:** 10.3389/fpsyg.2025.1655405

**Published:** 2025-11-04

**Authors:** Maren Nyer, Sofia Montinola, Samantha Pegg, Yousif Alsaadi, Zainab O. Soetan, Simmie Foster, Dustin J. Rabideau, Juliana Peacock, Yian Wu, Anna Kinnas, David Mischoulon, Louisa G. Sylvia

**Affiliations:** ^1^Depression Clinical and Research Program, Massachusetts General Hospital, Boston, MA, United States; ^2^Department of Psychiatry, Harvard Medical School, Boston, MA, United States; ^3^Dauten Family Center for Bipolar Treatment Innovation, Massachusetts General Hospital, Boston, MA, United States; ^4^Biostatistics, Massachusetts General Hospital, Boston, MA, United States; ^5^Department of Medicine, Harvard Medical School, Boston, MA, United States

**Keywords:** yoga, heated yoga, survey, depression, facilitators, barriers, mental health

## Abstract

The present study surveyed predominantly heated yoga users (*N* = 2,514) from CorePower Yoga, the largest U.S. yoga studio brand, to explore: (1) demographic characteristics and (2) facilitators and barriers to class participation. The sample was predominantly white (78%), at least 4-year college educated (90%), and female (87%). Twenty-three percent of participants (*n* = 587) self-reported being diagnosed with clinical depression. Most participants practiced yoga several days a week, and for at least 2 years, predominantly heated yoga classes. Out of a list of options, participants selected facilitators to heated yoga practice and barriers to yoga practice. Physical and mental health benefits of heated yoga facilitated their practice, whereas being around others, improved sleep, and reduction of physical pain were ranked as the lowest facilitators. Scheduling and expense related concerns were the highest ranked barriers to yoga classes, while not liking class participants, administrative issues, and discomfort with exercising around others were the lowest ranked barriers. Overall, these barriers and facilitators were generally consistent across subgroups (e.g., age, gender, race, ethnicity). Participants with self-reported diagnosed clinical depression reported improvement in mood as a more important facilitator than improvement in physical health, and barriers were consistent with the general survey population. Further research is needed to characterize facilitators and barriers to practice and strategies for improved usage.

## 1 Introduction

The rates of individuals practicing yoga in the U.S. are increasing. According to the 2022 National Health Interview Survey (NHIS), which examined trends in complementary medicine use over a 20-year period, the percentage of U.S. adults practicing yoga tripled from 5% in 2002 to 15.8% in 2022 (NCCIH, 2022). In the U.S., heated yoga originated in the 1970s with the introduction of Bikram yoga, a standardized sequence of 26 postures and two breathing exercises practiced in a heated environment (Fish, 2006; Singleton, 2010). After that, other forms of heated yoga were established in the U.S., such as CorePower Yoga, Baptiste Power Yoga, and heated vinyasa flow. To date, there are no known published reports specifically on heated yoga practice rates in the U.S.

In terms of the mental health benefits of heated yoga, studies have shown (1) improvements in self-reported and clinician-rated depressive symptoms in those with depression (Nyer et al., 2019, 2023; La Rocque et al., 2021); (2) reductions in cortisol reactivity and self-reported measures of binge and affective eating in women at risk for obesity-related illness (Hopkins et al., 2016); (3) improvements in wellbeing, life satisfaction, general health, mindfulness, peace of mind, and eudaimonic wellbeing in healthy yoga-naïve adults (Hui et al., 2022); (4) improvements in mindfulness, perceived stress, cardiorespiratory endurance, flexibility, and balance in healthy yoga-naïve adults (Hewett et al., 2011); and (5) improvements in self-efficacy, perceived stress, quality of life, general health, and energy/fatigue in sedentary, stressed adults (Hewett et al., 2018).

Heated yoga may also improve physical health. Heated yoga has been associated with cardiovascular fitness in terms of (1) improving arterial stiffness in overweight/obese participants (Hewett et al., 2018); (2) improving arterial stiffness in young adults (Hunter et al., 2013); and (3) improving vascular endothelial function in middle and older-aged adults (Hunter et al., 2017). Heated yoga in young healthy adults has been associated with improvements in measures of musculoskeletal fitness, such as deadlift strength, lower back/hamstring flexibility, and shoulder flexibility, and modestly decreased body fat compared to controls (Tracy and Hart, 2013). Additionally, heated yoga has been associated with increased aerobic fitness when compared to normal temperature yoga, with some evidence for cellular thermotolerance adaptations (Bourbeau et al., 2021).

These mental and physical health benefits could be related to each of the three main components of heated yoga, namely exercise, heat, and yoga. For example, one study that explored the contribution of yoga when compared to walking for those with depression in a 12-week trial, found that Iyengar yoga was associated with greater mood improvement and reduction in anxiety compared to a metabolically matched walking control condition (Streeter et al., 2010). Heat may also play a unique role; in a study in which heated yoga users performed the same posture sequence in heated and non-heated conditions, only the heated group showed acute IL-6 elevations (Lambert et al., 2020). This could potentially indicate that the heat may be associated with mental health benefits given that acute IL-6 spikes have been linked to antidepressant effects in other contexts [e.g., following whole-body hyperthermia (Flux et al., 2023)]. Finally, in women with depression, both heated yoga and aerobic exercise outperformed a waitlist control condition in reducing depressive symptoms, though the two exercise conditions did not differ from each other likely due to small sample size and limited power (La Rocque et al., 2021).

There remains a lack of targeted research exploring the demographics, practice patterns, and motivations of heated yoga users. This gap is important, as understanding who participates in heated yoga—and why—can inform more accessible and effective interventions. To address this, the present study surveyed the characteristics of predominantly heated yoga users, as well as facilitators and barriers to practice, across CorePower Yoga studios—the largest yoga studio brand in the U.S.

## 2 Method

### 2.1 Procedure

All study procedures were approved by the Mass General Brigham Institutional Review Board. An email inviting users of CorePower Yoga to complete a public survey link was distributed through CorePower Yoga's commercial mailing lists on November 21, 2024. The email was sent to a targeted group of users who visited CorePower Yoga studios at least twice in 2024 and had opted into email marketing, which was a total of 225,872 student profiles. Individuals answered screening questions to assess eligibility—i.e., 18 years or older, not currently pregnant, able to read and understand English, and users of CorePower Yoga. Eligible participants were then presented with a brief description of the study, contact information of study staff, and then the survey. The survey was designed to take approximately 5–10 min and consisted of questions related to participant demographics, non-heated and heated yoga experience, whether participants had ever been diagnosed with clinical depression, and facilitators and barriers to practicing classes at CorePower Yoga. Upon completion of the survey, participants were thanked for their time. Participants did not receive compensation for completing the survey. See Supplementary Figure 1 for the full survey questions.

### 2.2 CorePower Yoga

CorePower Yoga is the largest yoga studio brand (by studio count) in the U.S. with over 220 studios nationwide and offers a range of classes with varying heat, intensity levels, and duration. Its predominant and most popular formats are heated yoga classes and a heated “Yoga Sculpt” class practiced with light weights. The temperatures range from non-heated (residual heat can be present) to 105°F. According to CorePower Yoga, their most popular yoga class is a 60-min heated Power Yoga or “vinyasa” class practiced in 95°F called CorePower 2.

### 2.3 Data analysis

For the facilitator analysis, participants were analyzed if they provided at least one facilitator. For the barrier analysis, participants were analyzed if they provided at least one facilitator and at least one barrier. Descriptive statistics were reported for participant demographics and yoga experience and preferences, including facilitators and barriers. Participants also chose their top five facilitators out of 9 options (including “other”) to practicing heated yoga. Participants also chose their top five barriers to practicing yoga classes at CorePower Yoga out of 18 options. Facilitators and barriers were ranked by importance (1 = most important facilitator/barrier; 5 = least important facilitator/barrier). Please see Supplementary Figure 1 for the full survey for an exhaustive list of all facilitators and barriers. Facilitators were scored 6—chosen rank (i.e., on a scale from 0 to 5). Specifically, facilitators ranked as most important (i.e., first on the survey) were given the score of 5 for the participant. Facilitators ranked second most important were given the score of 4. This continued to the facilitator ranked least important out of 5 (i.e., their 5th choice), which was given a score of 1. If a participant did not rank a facilitator, the facilitator would get a score of 0. Scores for each facilitator were then averaged across participants. Higher mean scores indicate that more participants ranked that facilitator highly, while lower mean scores indicate that participants ranked the facilitator lower or did not rank the facilitator at all. The same scoring procedure was followed for barriers.

We also examined potential differences in reported facilitators and barriers based on demographic variables, depression diagnosis, years of experience, frequency of yoga practice, and heated vs. non-heated class preference (i.e., those who chose a heated yoga class as their first choice compared to those who chose a non-heated yoga class as their first choice). For some variables, categories were collapsed to examine differences in facilitators and barriers between two groups (see footnote^1^). We report on the three highest and three lowest ranked facilitators and barriers in these group-specific comparisons.

## 3 Results

### 3.1 Demographics

A total of 3,019 respondents were eligible, reviewed the study information sheet, and began the survey. There were 2,514 survey responses from participants who indicated at least one facilitator on the survey and included in study results. Participants were allowed to skip questions, thus, not all questions included responses from all participants. Participant demographics of the 2,514 participant sample are presented in Table 1. The median age of participants was 39 years. Most participants were female (87%) and White (78%). Regarding ethnicity, 11% of participants were Hispanic or Latino/a. Most participants had a household income above $125,000 (53%), were employed full-time (71%), and held a Bachelor's level degree or higher (90%). Regarding depression diagnosis, 23% reported having ever been diagnosed with clinical depression and 74% reported that they had not. Most participants reported practicing yoga (either heated or non-heated) for 5 years or more (64%), practicing more than 2 days per week (55%), and that practicing yoga improves their mental health (98%). Experience with yoga is reported in Table 2. Regarding heated yoga practice preference between heated vs. non-heated yoga, 87% selected a heated yoga class as their first choice of class, and 7% selected a non-heated yoga class as their first choice of class.

**Table 1:** Demographics of the sample.

**Variable (*n* = 2,514 responses)**	***n* (%)**
**Gender identity**	
Woman	2,186 (87)
Man	308 (12)
Non-binary/gender non-conforming	5 (<1)
Transgender woman	5 (<1)
Transgender man	1 (<1)
Prefer not to answer or missing	9 (<1)
**Race** ^a^	
White	1,969 (78)
East or Southeast Asian	163 (7)
Another race or national origin	100 (4)
African American, Black, West Indian, Afro-Caribbean, Afro-Latino/x, African (sub-Saharan)	57 (2)
South Asian	28 (1)
American Indian/Alaska Native	13 (<1)
Native Hawaiian or Other Pacific Islander	12 (<1)
West or Southwest Asian	6 (<1)
Multiple races selected	114 (5)
Prefer not to answer or missing	52 (2)
**Ethnicity**	
Not Hispanic or Latino/a	2,152 (86)
Hispanic or Latino/a	272 (11)
Prefer not to answer or missing	90 (4)
**Household income**	
$0–49,999	166 (7)
$50,000–74,999	213 (9)
$75,000–99,999	267 (11)
$100,000–124,999	284 (11)
$125,000–199,999	477 (19)
$200,000+	850 (34)
Prefer not to answer or missing	257 (10)
**Current employment status**	
Employed full-time (35+ h per week)	1,791 (71)
Employed part-time	252 (10)
Student	163 (7)
Retired	154 (6)
Primary household caregiver, full-time parent or childcare provider	57 (2)
Unemployed	52 (2)
On disability	3 (<1)
Prefer not to answer or missing	42 (2)
**Highest level of education**	
Less than high school diploma	2 (<1)
High school diploma or equivalent	39 (2)
Some college, but no degree	134 (5)
Associate's degree	73 (3)
Bachelor's degree	1,187 (47)
Master's degree	733 (29)
Professional or doctorate degree	331 (13)
Prefer not to answer or missing	15 (<1)
**Depression diagnosis**	
Yes	587 (23)
No	1,858 (74)
Prefer not to answer or missing	69 (3)

^a^Participants were allowed to choose multiple race categories. Individuals who selected more than one of the following were included in the “Multiple races selected” category: “White”, “East or Southeast Asian”, “Another race or national origin”, “African American, Black, West Indian, Afro-Caribbean, Afro-Latino/x, African (sub-Saharan)”, “South Asian”, “American Indian/Alaska Native”, “Native Hawaiian or Other Pacific Islander”, “West or Southwest Asian”. If a participant only selected one category, that is the category they show up in in the above table.

**Table 2:** Yoga experience of the sample.

**Variable (*n* = 2,514 responses)**	***n* (%)**
**Years practicing yoga**	
<1 year	301 (12)
2–4 years	590 (24)
5–10 years	686 (27)
More than 10 years	913 (36)
Prefer not to answer or missing	24 (1)
**Frequency of yoga practice**	
Not at all	8 (<1)
A few times per month	334 (13)
1–2 days per week	755 (30)
3–4 days per week	991 (39)
5–7 days per week	395 (16)
Prefer not to answer or missing	31 (1)
**Does practicing yoga improve your mental health?**	
Yes	2,474 (98)
No	16 (<1)
Prefer not to answer or missing	24 (1)

### 3.2 Barriers and facilitators to yoga participation

Ranked facilitators to heated yoga participation and barriers to yoga classes at CorePower Yoga across the sample are presented in Table 3.

**Table 3:** Facilitators to heated yoga and barriers to yoga classes at corepower yoga across the sample.

	***M***		
**Facilitators to heated yoga**	**Overall sample (*****n*** = **2,514)**	**Depression diagnosis (*****n*** = **587)**	**No depression diagnosis (*****n*** = **1,858)**
I want to become more flexible, fit or strong	2.88	2.50^*^	3.02
To improve my physical health	2.67	2.41^*^	2.76
I feel less stressed or anxious after I practice	2.49	2.73^*^	2.40
It helps improve my mood (less depressed, sad or down)	2.13	2.53^*^	2.00
I want to lose weight	1.18	1.24	1.17
To relieve physical pain	0.95	0.86^*^	0.97
It helps me to sleep better	0.90	0.92^*^	0.90
I like to be around other people	0.73	0.69	0.74
Other	0.12	0.10	0.13
**Barriers to yoga at CorePower Yoga**	**Overall sample (*****n*** = **1,323)**	**Depression diagnosis (*****n*** = **342)**	**No depression diagnosis (*****n*** = **938)**
The class schedules are not offered at convenient times for me	1.89	2.00	1.84
The classes are too expensive for me	1.77	1.78	1.78
It doesn't fit well with my schedule	1.46	1.45	1.47
The classes are too crowded	1.13	1.08	1.14
It's not convenient for me to get to CorePower Yoga	0.82	0.75^*^	0.85
The types of yoga/classes I want are not provided	0.69	0.78^*^	0.65
Teacher quality below expectations	0.52	0.50^*^	0.53
The studio is not nice enough (e.g., not enough cubbies, bathrooms, showers?)	0.47	0.56^*^	0.44
My financial situation changed	0.41	0.42	0.40
I no longer live or work near a studio	0.36	0.36^*^	0.37
An injury, pregnancy or another condition is preventing me from practicing	0.36	0.39^*^	0.34
The classes are too easy for me	0.25	0.25	0.25
I can't get childcare	0.24	0.23^*^	0.23
The classes are too hard for me	0.20	0.18^*^	0.20
I don't feel like I belong at CorePower Yoga	0.18	0.23^*^	0.16
I do not like exercising around other people/I feel self-conscious	0.12	0.18	0.10^*^
I had billing or account issues	0.11	0.11	0.11^*^
I do not like the other class participants	0.08	0.09	0.08

M represents mean scores where higher scores suggest that more participants ranked the item and tended to rank the item higher. Lower scores suggest that participants ranked the item lower or did not rank it at all. ^*^The order between depression diagnosis subgroups and overall sample was not the same for these items via visual comparison of ranked order lists.

#### 3.2.1 Facilitators

As reported in Table 3, across the sample of 2,514 respondents, the highest three ranked facilitators (in order of highest ranked to lowest ranked) to taking heated yoga classes were: (1) “I want to become more flexible, fit or strong”; (2) “To improve my physical health”; and (3) “I feel less stressed or anxious after I practice”. The three highest ranked facilitators differed for those with a depression diagnosis (*n* = 587): (1) “I feel less stressed or anxious after I practice”; (2) “It helps improve my mood (less depressed, sad or down)”; and (3) and “I want to become more flexible, fit, or strong” (see Table 3). For participants without a depression diagnosis (*n* = 1,858), the highest three ranked facilitators and their order did not differ from the overall sample. The highest three ranked facilitators generally did not differ by gender, race, ethnicity, age, income, years of yoga experience, yoga practice frequency, and yoga class preference, although the order varied in some cases.

Across the sample of 2,514 respondents, the lowest three ranked facilitators (in order of least ranked/lowest on the list to higher ranked) (besides an open-ended “other” response) were: (1) “I like to be around other people”; (2) “It helps me sleep better”; and (3) “To relieve physical pain”. These differed by age, years of yoga experience, and yoga class preference. Specifically, the lowest ranked facilitator was “I want to lose weight” for participants who were 39 years or older, those with 5 or more years of yoga experience, and those who preferred non-heated yoga as their first choice (vs. heated yoga classes) (see Table 3). The lowest three ranked facilitators generally did not differ by gender, race, ethnicity, income, yoga practice frequency, and depression diagnosis, although the order varied in some cases.

#### 3.2.2 Barriers

Across the sample of 1,323 respondents included in barrier analyses (i.e., those who provided at least one facilitator and at least one barrier), the highest three ranked barriers (out of the 18 options available) for taking yoga classes at CorePower Yoga were: (1) “The class schedules are not offered at convenient times for me”; (2) “The classes are too expensive for me”; and (3) “It doesn't fit well with my schedule”. These barriers differed by age, income, years of yoga experience, and preference for heated yoga classes. Specifically, the third highest ranked barrier was “The classes are too crowded” for participants younger than 39 years old, with an income less than $125,000, and with less than 5 years of yoga experience. The highest three ranked barriers generally did not differ by gender, race, ethnicity, yoga practice frequency, yoga class preference, and depression diagnosis, although the order varied in some cases (see Table 3).

Across the sample of 1,323 respondents included in barrier analyses, the lowest three ranked barriers (out of the 18 available options; in order of least ranked/lowest on the list to higher ranked) were: (1)“I do not like other class participants”; (2) “I had billing or account issues”; and (3) “I do not like exercising around other people/I feel self-conscious”. These differed by gender, ethnicity, age, income, years of yoga experience, and yoga class preference (see Table 3). Specifically, the third lowest ranked barrier was “I can't get childcare” for men and participants who preferred non-heated yoga as their first choice (vs. heated yoga classes). This barrier was also the second lowest ranked barrier for participants who were younger than 39 years old, had an income less than $125,000, and had less than 5 years of yoga experience. The second lowest ranked barrier was “An injury, pregnancy, or another condition is preventing me from practicing” for participants who identified as Hispanic or Latino/a. The lowest three ranked barriers generally did not differ by race, yoga practice frequency, and depression diagnosis, although the order varied in some cases.

## 4 Discussion

The present study examined participant demographics, as well as facilitators of and barriers to predominantly heated yoga participation, among users of CorePower Yoga, the largest brand of community-based yoga studios in the U.S. Among the 2,514 participants who ranked at least one facilitator, the majority of respondents (87%) selected a heated yoga class as their first-choice class. This was expected, given that most classes offered at CorePower Yoga are heated.

Regarding demographic characteristics of this predominantly heated yoga sample, the majority of participants were female, white, and highly educated, and reported higher income levels. This is consistent with multiple reports that have demonstrated that the typical yoga users in the U.S. are female, college educated, younger, and white (Birdee et al., 2008; Zhang et al., 2021; Yoga Among Adults Age 18 Older: United States, 2022) and with higher incomes (Cramer et al., 2016). These studies did not differentiate heated and non-heated yoga. The only known larger study of heated yoga (*N* = 700) that reported on demographic characteristics also reported a predominantly female (60%) and white (78%) sample (Firebaugh and Eggleston, 2017). Our study had a median age of 39 years, and the above study had a similar age range with the average age of 32.6 years (Firebaugh and Eggleston, 2017).

Regarding yoga use, the majority reported practicing yoga for at least 2 years, at least several days per week. Additionally, the email link to the survey was sent to a group of CorePower Yoga users who visited their studios at least twice in the year the survey was distributed. It is possible that within this group that those who care most about their practice at CorePower Yoga were more likely to take the time to fill out this uncompensated survey. Thus, this may not be a complete reflection of the overall populations of yoga users at CorePower Yoga.

Across the general sample, the highest ranked facilitators to participating in heated yoga classes were related to improving physical health and fitness as well as reducing stress or anxiety. These results may suggest that participants are interested in the physical and mental health benefits of heated yoga as described in the literature (Hunter et al., 2016, 2013, 2017; Tracy and Hart, 2013; Lambert et al., 2020; Hewett et al., 2015). These highly ranked facilitators of heated yoga were generally consistent between subgroups of participants, with one notable exception being participants who had been diagnosed with clinical depression. These participants identified reducing stress or anxiety and improving mood as higher ranked facilitators—i.e., mental health facilitators were ranked higher than physical health in the depressed sample when compared to the non-depressed sample. This, too, aligns with previous work identifying psychological benefits of heated yoga for depressive symptoms (Nyer et al., 2019, 2023; La Rocque et al., 2021) and perceived stress and cortisol reactivity in the face of a lab-based stressor (Hopkins et al., 2016).

The lowest ranked facilitators to participating in heated yoga classes (i.e., factors that were ranked the lowest if at all) across the sample were being around others, helping with sleep, and relieving physical pain. This was a surprising finding, as prior literature has shown that heated yoga (Kudesia and Bianchi, 2012), and to a much greater extent non-heated yoga (Turmel et al., 2022; Zhu et al., 2023; Wang et al., 2020; Bankar et al., 2013; Alghosi et al., 2025), may provide benefits for sleep. In addition, non-heated yoga has also been shown to reduce pain in chronic pain conditions (Wren et al., 2011; Büssing et al., 2012; Anheyer et al., 2022). It is possible, given that the study did not specifically recruit a population with physical pain or sleep concerns, that the benefits may not be noticeable for this general population.

The highest ranked barriers to participating in yoga at CorePower Yoga across the sample were related to class scheduling conflicts and expense. These ranked barriers differed slightly for those who were comparatively younger, had lower income, and had less yoga experience. Specifically, these subgroups ranked that the classes were too crowded as a barrier more so than the schedule-related concerns. It is possible that post the COVID-19 pandemic, there are concerns with infection in crowded enclosed spaces. These results highlight the importance of having many classes across different days and times to increase access and reduce crowdedness in classes. In addition, yoga classes are expensive for many. There are many classes available online; however, heated yoga is difficult to do at home without a heated space, and thus, is only accessible to those who live near heated studios. There are no trials to date comparing heated and non-heated yoga directly for mental health benefits. If heated yoga is associated with greater effectiveness in large-scale trials, accessibility for lower income populations and rural populations or those in places without heated yoga studios will need to be considered. For example, if it is determined that there is a need for heated yoga in lower income communities, one option could be that heated yoga could be added to YMCAs or other community centers to increase access. Considerations for making yoga more accessible to lower income populations can include things like location, cost, and childcare (Nagaswami et al., 2024). Additional research is needed to understand who is more likely to attend and benefit from heated yoga.

There are a several limitations of the present study worth noting. Responses were limited to options and questions included in the survey, and participants were not required to answer each question or complete all rankings. While this reduced burden and allowed participants to respond to questions more comfortably, response rates varied across questions. The response rate was particularly low for barriers. This could also be due to the order of survey questions, as barriers were amongst the last questions on the survey, or it is possible that participants may not have identified with the barrier choices listed in the survey and opted not to respond. Since the survey was accessible via a public link, there is the possibility that the same individual could have taken the survey multiple times. However, this is unlikely as there was no renumeration offered for the survey and therefore no benefit to producing multiple entries. The survey did not separate heated and non-heated yoga in some of the questions—e.g., the survey listed facilitators of “heated yoga” and barriers to “yoga”, which was an oversight in survey design. There are likely several sources of selection bias in this data. This is only a survey of CorePower Yoga users and also only a subsample of those who filled out the survey. This means that they are (1) likely highly motivated, and (2) have the time to fill out the survey. The sample had notably higher income, which could suggest that CorePower Yoga serves a higher income population and/or those with higher incomes had more leisure time to participate in the survey. Thus, the information on barriers and benefits of heated yoga may not be generalizable to lower income population. Special considerations may be needed for low-income populations (see Nagaswami et al., 2024 for a review). The participants in the survey also may be more invested in this form of yoga as their survey responses reflected that they practiced yoga for at least 2 years and more than 2 days per week.

Future research using qualitative methods is needed to more fully improve our understanding of participant experiences, including facilitators, barriers, and benefits/risks of practice. Furthermore, participants were all recruited only through CorePower Yoga studios in the U.S., and thus, the sample cannot be considered representative of all yoga users generally in the U.S. Although the sample, and the community from which it was drawn, was quite large and is a strength of this study, we had a low response rate (i.e., < 5% of CorePower Yoga patrons), suggesting possible selection bias. Future research could survey participants from other heated yoga studios outside of the CorePower Yoga network and pay participants to respond, thus improving the generalizability of the sample. Finally, responses were based on self-report, including lifetime clinical depression diagnosis, which would ideally be confirmed via clinician-rated assessment. Larger scale studies with more rigorous diagnostic methods are needed to more fully explore implications of depression and potential influence of comorbidities, such as sleep disorders and chronic pain conditions, in results. Additionally, as this is a group who practices predominantly a relatively rigorous form of heated yoga frequently and for a long period of time, they may be healthier on average than the general population, and as such, heated yoga as an intervention for specific conditions cannot be drawn from this sample.

Despite these limitations, the present study is the first known large-scale survey of predominantly heated-yoga users. As such, it adds to the literature-base in characterizing the sample and also identifies facilitators and barriers for a sample of predominantly heated yoga users. As heated yoga use increases in the U.S., the present study represents a first step in examining experiences and motivations of heated yoga users. Additional research building on this work has the potential to impact the development of interventions using heated yoga to improve physical and mental health.

## Data Availability

The raw data supporting the conclusions of this article will be made available by the authors, without undue reservation.

## Footnotes

^1^Gender was collapsed into men (including transgender men; facilitator *n* = 309; barrier *n* = 122) and women (including transgender women; facilitator *n* = 2,191; barrier *n* = 1,194). Race was collapsed into White (facilitator *n* = 1,969; barrier *n* = 985) and any other race (facilitator *n* = 376; barrier *n* = 238). Ethnicity was defined as Hispanic or Latino/a (facilitator *n* = 272; barrier *n* = 158) and not (facilitator *n* = 2,152; barrier *n* = 1,122). Age was divided into those who were younger than the median age of 39 (facilitator *n* = 1,162; barrier *n* = 718), and those who were at least 39 (facilitator *n* = 1,263; barrier *n* = 551). Income was collapsed into annual household income less than $125,000 (facilitator *n* = 930; barrier *n* = 551) and at least $125,000 (facilitator *n* = 1,327; barrier *n* = 658). Years of yoga practice experience were collapsed into less than 5 years of experience (facilitator *n* = 891; barrier *n* = 499) and 5 or more years of experience (facilitator *n* = 1,599; barrier *n* = 811). Yoga frequency was divided into 0-2 times per week (facilitator *n* = 1,097; barrier *n* = 698) and more than 2 times a week (facilitator *n* = 1,386; barrier *n* = 611). Yoga class preference was divided into those who chose heated yoga as their first choice (facilitator *n* = 2,196; barrier *n* = 1,126) and those who chose non-heated yoga as their first choice (facilitator *n* = 182; barrier *n* = 127). Depression diagnosis history was defined as having a self-reported diagnosis (facilitator *n* = 587; barrier *n* = 342) and not (facilitator *n* = 1,858; barrier *n* = 938).
