# Supplementary material for: Demographics, facilitators, and barriers among predominantly heated yoga users: a survey of the largest U.S. yoga studio brand
Source: Front Psychol. 2025 Nov 4;16:1655405. doi: 10.3389/fpsyg.2025.1655405 (PMC12623406; doi:10.3389/fpsyg.2025.1655405)
Supplement: Supplementary file 1 [file Supplementary_file_1.docx]

**Demographics, Facilitators, and Barriers Among Predominantly Heated Yoga Practitioners: A Survey of the Largest U.S. Yoga Studio Brand**

**Supplementary Material**

**Supplementary Material 1**

*Survey Questions*

**Pre-Screener:**

1. Are you 18 or over? (Yes/No)

2. Are you currently pregnant? (Yes/No)

**Heated Yoga Survey:**

*Demographics Questions*

1. What is your current gender identity?

- Man
- Woman
- Transgender woman
- Transgender man
- Nonbinary/gender non-confirming
- Another gender identity
- I prefer not to answer

2. Please indicate your racial group (Select as many as apply)

- American Indian / Alaska Native
- African American, Black, West Indian, Afro-Caribbean, Afro-Latino/x, African (sub-Saharan)
- Asian - East or Southeast
- Asian - West or Southwest
- Asian - South
- White
- Native Hawaiian, Other Pacific Islander
- Another race or national origin
- I prefer not to answer

3. What is your ethnicity?

- Hispanic or Latino/a
- Not Hispanic or Latino/a
- I prefer not to answer

4. What is your current age? [Range: 18-100 or “I prefer not to answer”]

5. Over the past year, what was the total income of your household? *(By this we mean, how much money did anyone over the age of 15 contribute to the household? This includes paychecks, disability payments, government benefits, child support, social security, unemployment, or other similar financial assistance.)*

- 0-$49,999
- $50,000-$74,999
- $75,000-$99,999
- $100,000-$124,999
- $125,000-$199,999
- $200,000+
- I prefer not to answer

6. Employment:

- Employed full-time (35+ hours/per week)
- Employed part-time
- Student
- Unemployed
- On disability
- Primary household caregiver, full-time parent or childcare provider
- Retired
- I prefer not to answer

7. Education:

- Never attended school
- Less than High School diploma
- High School diploma or equivalent
- Some college, but no degree
- Associate’s degree
- Bachelor’s degree
- Master’s degree
- Professional or Doctorate degree
- I prefer not to answer

*Yoga Questions*

1. How long have you been practicing yoga?

- < 1 year
- 2-4 years
- 5-10 years
- More than 10 years

2. How often do you practice yoga?

- Not at all
- A few times per month
- 1-2 days/week
- 3-4 days/week
- 5-7 days/week

3. Which CorePower Yoga class do you attend most often? Rank your top 3.

- CorePower Yoga 1 (C1)
- CorePower Yoga 2 (C2)
- Yoga Sculpt
- Hot Power Fusion
- Hot Yoga
- CoreRestore

4. Does practicing yoga improve your mental health?

- Yes
- No
- I prefer not to answer

5. Have you ever been diagnosed with clinical depression?

- Yes
- No
- I prefer not to answer

6. Why do you practice heated yoga? Rank your top 5 in order of importance (1 = most important).

- I like to be around other people
- I want to lose weight
- I want to become more flexible, fit or strong
- It helps me to sleep better
- I feel less stressed or anxious after I practice
- It helps improve my mood (less depressed, sad or down)
- To relieve physical pain
- To improve my physical health
- Other (please specify): (Select and write-in)

7. Are there any barriers or challenges to taking yoga classes at CorePower Yoga (CPY)? (Yes/No)

8. If yes, rank your top 5 in order of importance (1 = most important).

- The class schedules are not offered at convenient times for me
- It doesn’t fit well with my schedule
- It’s not convenient for me to get to CorePower Yoga
- The classes are too hard for me
- The classes are too easy for me
- An injury, pregnancy or another condition is preventing me from practicing
- The types of yoga/classes I want are not provided
- I do not like the other class participants
- I had billing or account issues
- I no longer live or work near a studio
- I can’t get childcare
- The classes are too expensive for me
- I do not like exercising around other people/I feel self-conscious
- My financial situation changed
- Teacher quality below expectations
- The classes are too crowded
- I don’t feel like I belong at CorePower Yoga
- The studio is not nice enough (e.g., not enough cubbies, bathrooms, showers?)

**Supplementary Material 2**

*Information on CorePower Yoga Classes*

| Class Title | Class Description | Physical Intensity | Temperature with humidifier | Temperature without humidifier |
| --- | --- | --- | --- | --- |
| CorePower 1 | Explore the fundamental principles and postures of Vinyasa yoga with no added heat. | Low-med | 85°F/humidity off | 88°F |
| CorePower 2 | Turn stress into sweat as you move through more challenging postures. | Med-high | 95°F/40% | 98°F |
| Yoga Sculpt | Yoga, cardio and strength moves boost metabolism and build lean muscle. | High | 92°F/humidity off | 95°F |
| Hot Power Fusion | Where the meditative qualities of hot yoga and the intensity of power yoga meet. | Mid | 103°F/40% | 105°F |
| Hot Yoga | Amplify your practice in this high heat and humidity class designed to build towards several peak postures | Mid | 105°F/40% | 108°F |
| CoreRestore | Reboot and rebuild with postures focused on stretching and breathing with no added heat. | Low | 75°F /humidity off | 78°F |

*Note*: This information was not provided to participants in the survey. This description provides heating guidelines for each class.
